# The “Spiked Helmet” Sign Associated with ST-Elevation Myocardial Infarction: A Case Report

**DOI:** 10.5811/cpcem.2021.1.50921

**Published:** 2021-05-10

**Authors:** Bruno Minotti, Jörg Scheler, Robert Sieber, Eva Scheler

**Affiliations:** * Cantonal Hospital of St. Gallen, Department of Emergency Medicine, St. Gallen, Switzerland; † Cantonal Hospital of St. Gallen, Department of Cardiology, St. Gallen, Switzerland

**Keywords:** spiked helmet sign, upper abdomen pain, chest pain, STEMI, emergency department, case report

## Abstract

**Introduction:**

The “spiked helmet” sign was first described in 2011 by Littmann and Monroe in a case series of eight patients. This sign is characterized by an ST-elevation atypically with the upward shift starting before the onset of the QRS complex. Nowadays the sign is associated with critical non-cardiac illness.

**Case Report:**

An 84-year-old man with a history of three-vessel disease presented to the emergency department with intermittent pain in the upper abdomen. The electrocardiogram revealed the “spiked helmet” sign. After ruling out non-cardiac conditions the catherization lab was activated. The coronary angiography revealed an acute occlusion of the right coronary artery, which was balloon-dilated followed by angioplasty. The first 24 hours went uneventfully with resolution of the “spiked helmet” sign. On the second day, however, the patient died suddenly and unexpectedly.

**Conclusion:**

Despite the association with non-cardiac illness, the “spiked helmet” sign can be seen by an acute coronary artery occlusion as an ST-elevation myocardial infarction (STEMI). Reciprocal ST-depression in these cases should raise the suspicion of STEMI.

## INTRODUCTION

In 2011 Littmann and Monroe described for the first time a particular electrocardiogram (ECG) sign in a case series of eight patients.^1^ This sign has been characterized by an ST-elevation with atypically upward shift starting before the onset of the QRS complex. None of these patients had critical cardiac illness. Acute myocardial infarction was ruled out by cardiac serum markers. The sign has been associated with a rapid increase in thoracic or intra-abdominal pressure.^2^ Association with confirmed coronary artery occlusion has never been reported.

## CASE REPORT

**Image 1:**
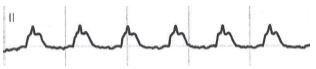
Electrocardiographic (lead II) taken from the Monitor System demonstrating the “spiked helmet” sign.

An 84-year-old man was brought to our emergency department by ambulance with intermittent pain in the upper abdomen and shortness of breath for three hours. He had a history of coronary three-vessel disease (first diagnosis 2004) with prediabetes, hypertension, hyperlipidemia, and past history of active smoking. Vital signs were in the normal range. Rapid first clinical assessment was unremarkable. The first ECG was seen on the monitor in lead II (Image 1).

**Image 2:**
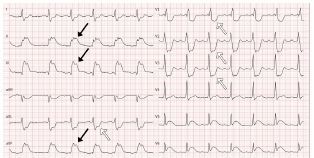
12-lead electrocardiogram at the presentation demonstrating the “spiked helmet” sign (black arrows) with reciprocal changes (white arrows).

The ECG “strip” showed sinus rhythm at 76 beats per minute with apparent ST-segment elevation, but with the upward shift starting before the onset of the QRS complex. This pattern was consistent with the “spiked helmet” sign. Lung auscultation showed bilateral vesicular breath sounds. Abdomen was soft and nontender with reduced peristalsis. Focused point-of-care ultrasound was performed with ubiquitous pleural sliding excluding a large pneumothorax and abdominal examination ruling out free fluid and gastrointestinal distension. We obtained in parallel a 12-lead ECG (Image 2).

**Image 3:**
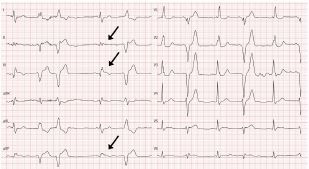
Post-interventional electrocardiogram with resolution of the “spiked helmet” sign (arrows).

The ECG showed a sinus rhythm at 78 beats per minute with first-degree atrioventricular block, right bundle branch block and ST-elevation in the inferior leads, again with the upward shift starting before the onset of the QRS complex. Due to the reciprocal ST-depression in lead I and aVL, the catherization lab was activated and the patient underwent coronary angiography. The cardiologist found an occlusion of the distal right coronary artery (RCA), which was the dominant vessel. The occlusion of the RCA was balloon-dilated followed by angioplasty. A post-interventional ECG with asymptomatic patient was obtained (Image 3).

The “spiked helmet” sign had resolved, with all that remained a nonspecific intraventricular block in the inferior leads and a slightly long corrected QT interval (QTc) of 480 milliseconds. The first 24 hours after intervention was uneventful. However, on the second day the patient had a fulminant collapse with hemodynamic instability. The patient had declared earlier to abstain from further intensive care therapy. Supportive therapy was performed and he died a few hours later.

**CPC-EM Capsule**

What do we already know about this clinical entity?

*The “spiked helmet” sign is characterized by an ST-Elevation with the upward shift starting before the onset of the QRS complex. This sign is associated with critical non-cardiac illness*.

What makes this presentation of disease reportable?

*We report the case of a patient presenting to the emergency department with chest pain and the “spiked helmet” sign on electrocardiogram (ECG). An acute coronary artery occlusion was responsible for the ECG changes*.

What is the major learning point?

*The “spiked helmet” sign can be seen by an acute coronary artery occlusion as an ST-elevation myocardial infarction (STEMI). Reciprocal ST-depression in these cases should raise the suspicion of STEMI*.

How might this improve emergency medicine practice?

*The “spiked helmet” sign can be seen in various diseases, including myocardial infarction. Differential diagnosis of ST-Elevation on ECG is broad and has to be scrutinized with care*.

## DISCUSSION

We report this case of a patient with the “spiked helmet” sign and an acute coronary artery occlusion with ST-elevation myocardial infarction. In the first case series by Littmann and Monroe, the “spiked helmet” sign was present exclusively in the inferior leads, as was noted in our case. The postulated mechanism of this phenomenon is an epidermal or diaphragmatic “stretch” due to an acute rise in the intrathoracic or intra-abdominal pressure. In fact, Tomcsany et al described the “spiked helmet” sign in one case of gastric distension and one case of pneumothorax.^2^ Littmann and Monroe also described a case of real-time recognition of the “spiked helmet” sign in a patient with pneumothorax.^3^ However, the sign has also been described in patients with subarachnoid hemorrhage and after ablation of the left stellate ganglion, in which an adrenergic excess was postulated as the mechanism associated with Takotsubo cardiomyopathy and long QT.^4^ Independent of the etiology, the “spiked helmet” sign has been associated with a very high mortality.^1^ We found only one report of a patient with the “spiked helmet” sign interpreted as a myocardial infarction, but the documentation is poor and coronary angiography was not performed.^5^ In our case we can clearly associate the “spiked helmet” sign to an acute major coronary artery occlusion resulting in myocardial infarction. A nonspecific intraventricular block in the inferior leads and a slightly long QTc could have played a role leading to this particular ECG configuration. The prognostic role of this sign in cardiac disease remains unclear.

Finally, we would like to emphasize that the definition of the “spiked helmet” sign is not unambiguous, because just defined as an ST-elevation with the upward shift starting before the onset of the QRS complex. A recent case of Crinion et al showed the “spiked helmet” sign by a patient with septic shock, where however the upward shift preceding the QRS complex could have been given by a negative T wave, and not necessarily by an elevation of the isoelectric line.^6^ Interobserver agreement should be investigated in future studies. The differential diagnoses for ST-elevations remain broad and should be scrutinized with attention. The history, physical exam, and clinical picture with possibly venous blood gas analysis with electrolytes, and point-of-care ultrasound could rapidly help to lead to the final diagnosis.

## CONCLUSION

Despite the association with non-cardiac illnesses, the “spiked helmet” sign can be seen in acute coronary artery occlusion as an ST-elevation myocardial infarction (STEMI). Reciprocal ST-depression in these cases should raise the suspicion of STEMI.
